# Correlation of Ultrasonographic Estimation of Fetal Weight with Actual Birth Weight as Seen in a Private Specialist Hospital in South East Nigeria

**DOI:** 10.1155/2019/3693797

**Published:** 2019-10-27

**Authors:** Chisolum Ogechukwu Okafor, Charles Ikechukwu Okafor, Ikechukwu Innocent Mbachu, Izuchukwu Christian Obionwu, Michael Echeta Aronu

**Affiliations:** ^1^Department of Radiology, Nnamdi Azikiwe University, The Light Specialist Hospital, Nnewi Campus, Nnewi, Nigeria; ^2^Department of Obstetrics and Gynaecology, Nnamdi Azikiwe University, The Light Specialist Hospital, NnewiCampus, Nnewi, Nigeria; ^3^Radiology Unit, The Light Specialist Hospital, Anambra State, Nnewi, Nigeria

## Abstract

**Background:**

Ultrasound estimation of fetal weight at term provides vital information for the skilled birth attendants to make decisions on the possible best route of delivery of the fetus. This is more pertinent in a setting where women book late for antenatal care.

**Aim and Objectives:**

The study evaluated the accuracy of estimation of fetal weight with ultrasound machine at term.

**Methods:**

This was a cross sectional study conducted at a private specialist hospital in Nigeria. A coded questionnaire was used to retrieve relevant information which included the last menstrual period, gestational age, parity, and birth weight. Other information obtained includes Ultrasound-delivery interval, maternal weight, and route of delivery. The ultrasound was used to estimate the fetal weight. The actual birth weight was determined using a digital baby weighing scale. The data were inputted into Microsoft excel and analyzed using STATA version 14. Statistical significance was considered at *p*-values less than 0.05. Measures of accuracy evaluated in the statistical analysis included mean error, mean absolute error, mean percentage error, and mean absolute percentage error. Pearson correlation was done between the estimated ultrasound fetal weight and the actual birth weight. The proportion of estimates within ±10% of actual birth weight was also determined.

**Result:**

A total of 170 pregnant women participated in the study. The mean maternal age was 30.77 years ± 5.54. The mean birth weight was 3.47 kg ± 0.47, while the mean estimated ultrasound weight was 3.43 kg ± 0.8. There was positive correlation between the ultrasound estimated weight and the actual birth weight. The mean ultrasound scan to delivery interval was 0.8 days (with range of 0–2 days). The study recorded a mean error of estimation of 41.17 grams and mean absolute error of 258.22 grams. The mean percentage error was 0.65%, while the mean absolute error of estimation was 7.56%. About 72.54% of the estimated weights were within 10% of the actual birth weight.

**Conclusion:**

The ultrasound estimated fetal weight correlated with the actual birth weight. Ultrasound estimation of fetal weight should be done when indicated to aid the clinician in making decisions concerning routes of delivery.

## 1. Introduction

In utero estimation of fetal weight is an important component of management of pregnancy. It provides valuable information which aids the physician/midwife to take informed decisions concerning the timing and route of delivery [1, 2]. It is very valuable in selection of patients for vaginal birth after caesarean section (VBA) and assisted breech delivery. Antepartum weight estimation is also an important tool in the monitoring and detection of intrauterine growth restriction and macrosomia [3, 4]. Thus, fetal weight is an independent risk factor for determining perinatal mortality.

Several methods of estimation have been described in the literature. These include clinical method and ultrasonographic estimation. [5, 6] Obstetrics ultrasound has remained the corner stone of fetal weight estimation. Studies have shown that combination of parameters including fetal femur length and biparietal diameter improves the accuracy of estimation [4]. The accuracy of ultrasonographic weight estimation is affected by many factors. Studies have shown poor predictive value at the extremes of weight (low birth weight and macrosomia). [7, 8] There are conflicting reports on the accuracy of ultrasound fetal weight estimation at term. [9– 11] However, scan delivery interval can also affect the accuracy of fetal weight estimation. This has been attributed to the rapid weight gain at term which varies from one fetus to another.

In setting where women book late or missed the opportunity of obstetrics ultrasound at earlier gestational age, it becomes imperative to offer them fetal weight estimation at term when there is an indication to estimate the fetal weight before delivery. Ultrasound estimation of fetal weight can also be a vital component of determination of routes of delivery in conditions like diabetes mellitus in pregnancy, breech delivery and borderline pelvis. This will help the clinicians and the patients to make informed decisions on the route of delivery. This study evaluated the accuracy of ultrasonographic estimation of fetal weight at term.

## 2. Subjects and Methods

### 2.1. Study Site

The study was done at The Light Specialist Hospital, Nnewi, which is a private Specialist hospital that handles majorly Obstetrics and Gyneacology patients. It also has a radiology unit manned by a consultant radiologist.

### 2.2. Study Design

This was a cross sectional study conducted from August 2016 to August 2018.

### 2.3. Study Population

The study population included singleton pregnancies at term (37 completed weeks to 42 weeks) that met the inclusion criteria and gave consent for the study. Subjects who met the inclusion criteria and gave consent were recruited for the study. A total of 170 pregnant women participated in the study.

Inclusion criteria were pregnant women at term with singleton pregnancies, who presented in latent phase of labour or were admitted for elective vaginal or abdominal delivery. Only subjects who gave informed consent wererecruited for the study.

Exclusion criteria included multiple pregnancy, congenital anomalies, intrauterine fetal death, intrauterine growth restriction and ultrasound delivery interval greater than 2 days. All participants consented to the study.

### 2.4. Procedure and Data Collection Techniques

The ultrasound records and patients' folders were used to retrieve relevant information which included the last menstrual period, gestational age, parity, gender of the neonate, estimated fetal weight, and the actual birth weight. Other information obtained included Ultrasound-delivery interval, maternal weight and route of delivery.

### 2.5. Procedure for the Ultrasound Fetal Weight Estimation

Ultrasound scans were done by a consultant radiologist with training in fetal ultrasound using a 3.5 MHz curvilinear transducer of Mindray Digital Ultrasound diagnostic imaging system (model DP 50 by Shenzhen Mindray Biomedical Electronics Co ltd, NASHEN Shenzhen 518057 People Republic of China). The fetal weight was calculated using Hadlock 3 formula comprising of ultrasonographic measurements of biparietal diameter (BPD), abdominal circumference (AC), and femur length (FL). The BPD was obtained at a level that showed a smooth symmetric head, a well-defined midline echo, thalami, the cavum septum pellucidum, and the third ventricle on a transverse image of the skull. The calipers were placed at the outer margin of the parietal boneto the inner margin of the opposite side of the parietal bone. The fetal AC was obtained using a transverse image measured at the level where theright and left portal veins were continuous with one another, appearing like a “J shape,” and the shortest length of the umbilical segment of the left portalvein was depicted [2]. The fetal stomach represented a secondary landmark and the vertebrae were at the horizontal plane. The ellipse of the electronic calipers were then fitted to the outer skin edge and used to measure the AC. For FL measurement, an iliac bone was identified and the transducer then maneuvered until the full length of the femur was visible and as horizontal as possible. FL is the distance between outer borders of the diaphysis of thefemoral bone.

The actual birth weight was determined using a digital baby scale (SECALENA model 354 by SecaGmBH and co 22089 Hamburg Germany).

### 2.6. Outcome Measures

The principal outcome measure was the accuracy of estimation of fetal weight by the use of Ultrasound machine.

### 2.7. Data Analysis

The data were inputted into Microsoft excel and analyzed using STATA version 13. Statistical significance was considered at *p*-values less than 0.05. Measures of accuracy evaluated in the statistical analysis include mean error, mean absolute error, mean percentage error, mean absolute percentage error, and the proportion of estimates within ±10% of actual birth weight. The spearman correlation between the estimated fetal weight and the actual weight was also determined and plotted on a two-way scatterplot.

## 3. Results

**Figure 1:**
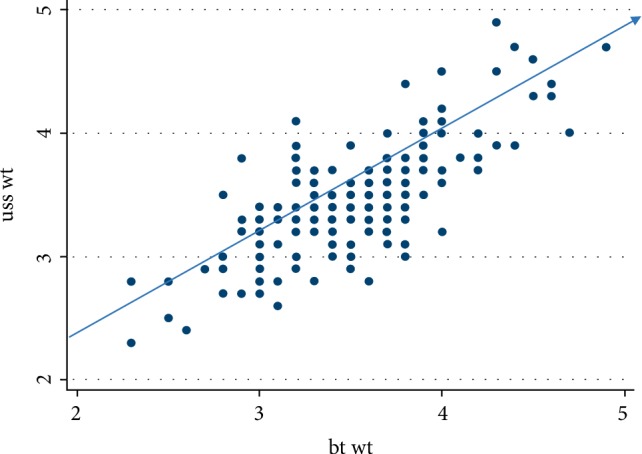
Correlation between the USS estimated weight and the actual birth weight. *R* = 0.75, *p*-value = 0.04.

A total of one hundred and seventy women participated in this study. The mean age of the women was 30.77 ± 5.54 years with a range of sixteen to forty-three years. The estimated mean fetal weight by Ultrasound was 3.43 ± 0.47 kg, while the mean birth weight was 3.48 ± 0.80 kg. There was no significant difference between the mean fetal weight estimated by Ultrasound scan and the mean birth weight (*p*-value = 0.8). 0nly 2 (1.18%) had low birth weight while macrosomia was recorded in 20 (11.76%). Majority of the neonates had normal birth weight 148 (87.06%).  Table 1 shows the summary statistics, while Figure 1 is the scatter diagram showing the correlation between estimated fetal weight by ultrasound and actual birth weight. There was a positive correlation between ultrasound estimated fetalweight and actual birth weight with Pearson's coefficient of 0.75 (*p*-value = 0.04).

The mean error in the estimation of birth weight was 41.0 g. The mean absolute error in the estimation of birth weight was 246.7 g. The mean percentage error for ultrasound estimated fetal weight was 1.9 ± 11.4%. This means that, in the overall study group, the ultrasonographic method slightly overestimated the actual birth weight. The mean absolute percentage error was 7.5 ± 5.89%.

In the study, the percentage of estimates within ±10% of the actual birth weight was found to be 72.94%. In 15.29% of the cases, ultrasound overestimated the birth weight and underestimated in 11.76%.  Table 2 shows accuracy of estimation while Table 3 shows the error of estimation.

## 4. Discussion

Our study was a cross sectional study involving 170 women with mean maternal age of 30.77. The mean birth weight was 3.47 kg while the mean estimated ultrasound weight was 3.43 kg. There was positive correlation between the ultrasound estimated weight and the actual birth weight. The mean ultrasound scan to delivery interval was 0.8 days (with range of 0–2 days). The study recorded a mean error of estimation of 41.17 grams and mean absolute error of 258.22 grams. The mean percentage error was 0.65%, while the mean absolute error of estimation was 7.56%. About 72.54% of the estimated weights were within 10% of the actual birth weight. The ultrasound over estimated the weight in 15.29% of the fetuses.

The mean birth weight of 3.47 kg is comparable to values from Lagos, Nigeria [10] but higher than values from Nepal [12] and Bangladesh [2]. The differences could be linked to the gestational age at scanning and the genetic and racial variations. There was no statistical difference between the estimated fetal weight and actual birth weight with a strong correlation between the ultrasound estimated weight and actual birth weight. This implies that when it is indicated, ultrasound should be used to estimate the fetal weight for the purposes of planning delivery and monitoring of the fetus. Similar findings have been observed by other studies [2, 6, 12].

The mean error of estimation of 41 grams was observed in our study. However, it should be interpreted with caution because it is the sum of both negative and positive values. The absolute mean error of estimation of 258.52 recorded in our study is comparable to observed values by Prasad et al in Nepal [12]. The mean absolute percentage of 7.56% is comparable to 8.76%, 7.2%, 7.7% reported by Prasad et al. [12], Lafont et al. [13], Colman et al. [14], and Houzé de l'Aulnoit et al. [15], respectively. It is also within the 6–12% reported in the literature [4].

The percentage of the estimated fetal weight within 10% of the actual birth weight was 72.94%. This is higher than values recorded by Prasad et al. (65%) [12], but similar to 72.25 and 75% percentage observed by Bolanka et al. and Colman et al., respectively. We estimated the fetal weight using the combination of abdominal circumference, femur length and biparietal diameter. Studies have shown that using more than one fetal parameter improves the accuracy of fetal weight estimation [2, 4]. This could be responsible for the observation in our study. Milner et al. in systematic review observed that using Hadlock 3 is associated with greater accuracy when compared to other methods [4].

The mean scan delivery interval in our study was 0.8 days. This could have also contributed to the concordance rate between the estimated fetal weight and the actual fetal weight observed in our study. The scan-delivery interval greater than seven days has been observed to reduce the accuracy of ultrasound estimation of fetal weight [4].

There has been major improvement in the use of ultrasound for fetal weight estimation since Kurjak and Breyer first reported their study using ultrasonic abdominometry in 1976 [16]. The improvement in the quality of ultrasound machine in the last decade coupled with improved training and use of multiple parameters have improved the fetal weight estimation by ultrasound machine. Thus, ultrasound will continue to play a vital role in the fetal weight estimation. This will help the clinician to make good decisions in the monitoring of the fetus and determination of the route of delivery.

One of the strengths of our study was that the entire ultrasound scan was done within 2 days of delivery. This has shown that ultrasound estimation of fetal weight can be done at term to help the patient and clinician to make informed decisions especially in evaluation of pregnant women with diabetes in pregnancy, suspected macrosomia and microsomia, breech presentation, and previous caesarean section. It is very pertinent in developing countries where they might not have assessed antenatal care.

Despite the obvious significance of the study, it has some limitations. Firstly. It is a single Centre study comprising mainly of homogenous group. Secondly, only two neonates had birth weight less than 2.5 kg, hence it will be difficult to make generalization with respect to low birth weight babies.

In conclusion, ultrasound machine has become a very vital tool in modern obstetrics practice. However, the reliability of the result depends a lot on the quality of the machine and skill of the sonographer.

## Figures and Tables

**Table 1:** Summary of the maternal and fetal variables.

Parameter	Mean (standard deviation)	Range	
		Maximum	Minimum
Maternal age	30.77 (5.54)	43	16
USS EFW	3.43 (0.47)	4.9	2.3
Actual birth weight	3.47 (0.48)	4. 9	2.3
USS—delivery interval (days)	0.85 (0.0.60)	2	0
Maternal weight	84.66 (13.61)	125	59

**Table 2:** Accuracy of method.

Parameter	Mean	SD
Mean error (grams)	41.17	32.8
Absolute mean error (grams)	258.82	201.35
Mean percentage error (%)	0.68	0.098
Absolute mean percentage error (%)	7.56	5.89
Accurate (within 10% of ABW)	72.94%	

**Table 3:** Error estimation.

Parameter	Number (%)
Accurate estimation	124 (72.94)
Inaccurate estimation	46 (27.06)
Overestimation	26 (15.29)
Underestimation	20 (11.77)

## Data Availability

The data will be made available on request.
